# Direct Copolymerization of CO_2_ and Diols

**DOI:** 10.1038/srep24038

**Published:** 2016-04-14

**Authors:** Masazumi Tamura, Kazuki Ito, Masayoshi Honda, Yoshinao Nakagawa, Hiroshi Sugimoto, Keiichi Tomishige

**Affiliations:** 1 Graduate School of Engineering, Tohoku University, Aoba 6-6-07, Aramaki, Aoba-ku, Sendai, 980-8579, Japan; 2 JST, PRESTO, 4-1-8, Honcho, Kawaguchi, Saitama, 332-0012, Japan; 3 Department of Industrial Chemistry, Faculty of Engineering, Tokyo University of Science, 12-1 Ichigaya-Funagawara, Shinjuku, Tokyo 162-0826 Japan

## Abstract

Direct polymerization of CO_2_ and diols is promising as a simple and environmental-benign method in place of conventional processes using high-cost and/or hazardous reagents such as phosgene, carbon monoxide and epoxides, however, there are no reports on the direct method due to the inertness of CO_2_ and severe equilibrium limitation of the reaction. Herein, we firstly substantiate the direct copolymerization of CO_2_ and diols using CeO_2_ catalyst and 2-cyanopyridine promotor, providing the alternating cooligomers in high diol-based yield (up to 99%) and selectivity (up to >99%). This catalyst system is applicable to various diols including linear C4-C10 α,ω-diols to provide high yields of the corresponding cooligomers, which cannot be obtained by well-known methods such as copolymerization of CO_2_ and cyclic ethers and ring-opening polymerization of cyclic carbonates. This process provides us a facile synthesis method for versatile polycarbonates from various diols and CO_2_ owing to simplicity of diols modification.

Direct transformation of CO_2_ to valuable chemicals is one of the hottest topics from the viewpoints of environmental and green chemistry^1^,^2^,^3^,^4^,^5^,^6^,^7^. Transformation of CO_2_ can be mainly categorized into two methods, reductive transformation and non-reductive transformation^1^,^4^,^5^. The non-reductive transformation of CO_2_ comprises the reactions of CO_2_ with compounds having polar functional groups such as alcohols and amines, providing various important chemicals such as ureas, carbamates, carbonates and polycarbonates, and it is promising because of lower energy input compared with the reductive transformation. However, since CO_2_ is very stable owing to the very strong double bond, an exquisite catalyst system to activate CO_2_ and reagents is essential. Carbonic anhydrase is well-known as an ideal catalyst system for non-reductive transformation of CO_2_, drastically accelerating the reaction of CO_2_ and H_2_O to bicarbonate and proton (~10^6^-fold vs non-catalyst)^8^,^9^,^10^,^11^,^12^. In this catalyst system, Zn^2+^ ion and a histidine residue activate H_2_O to generate reactive hydroxide species on Zn^2+^ ion (cooperation of Lewis acid and Lewis base), and CO_2_ is guided near the hydroxide species by the hydrophobic pocket composed of three valine residues (substrate concentration), forming a configuration that is conductive to reaction. As for artificial catalysts, achieving the sufficient level comparable to enzymes is quite difficult due to the smaller size of the artificial catalysts than enzymes. Therefore, it is desirable to create exquisite and precise artificial catalysts that can simultaneously activate CO_2_ and reagents in proximity of each other.

CeO_2_ has been widely used in the fields of catalyst and biological chemistry because of its unique acid-base and redox properties^13^,^14^, and has recently attracted much attention in liquid-phase organic syntheses at low temperature (≤473 K)^15^,^16^,^17^,^18^,^19^,^20^,^21^. In particular, it has been reported that CeO_2_ plays a crucial role in the catalytic non-reductive conversion of CO_2_ to organic carbonates, carbamates and ureas using alcohols or amines^22^,^23^,^24^,^25^,^26^,^27^,^28^,^29^,^30^,^31^,^32^, although these reactions have a common problem of the equilibrium limitation. Recently, we found that CeO_2_-catalyzed dehydration condensation of alcohols and CO_2_ in combination with CeO_2_-catalyzed hydration of 2-cyanopyridine to picolinamide enabled the formation of the corresponding organic carbonates in high yields^33^,^34^,^35^. The methanol-based yield of DMC in the reaction of CH_3_OH, CO_2_ and 2-cyanopyridine reached 94% yield, while the equilibrium yield of DMC is below 1% in the reaction of CH_3_OH and CO_2_ without 2-cyanopyridine. This is the first report on stoichiometric transformation of alcohols with CO_2_ to the corresponding carbonate. We also demonstrated that CO_2_ can be strongly adsorbed and activated on acid-base sites of CeO_2_^33^,^34^,^35^ and that methanol can be activated cooperatively by both CeO_2_ and 2-cyanopyridine at the interface between CeO_2_ and 2-cyanopyridine^36^, which has something common to the above enzyme catalyst system (mainly cooperation of Lewis acid and Lewis base, and substrate concentration). In addition, Urakawa and co-workers also applied this catalyst system to the DMC synthesis under a wide range of CO_2_ pressure (1–30 MPa) in a fixed bed reactor, achieving higher reaction rates than that in batch operation^37^. These results inspired us to apply this catalyst system to the direct synthesis of polycarbonates from CO_2_ and diols.

Polycarbonates has been commonly used as an engineering plastic, and the market size is 290 million ton/year in 2009 and expected to increase on average by about 4 ~ 6% each year to 2020^38^,^39^. Therefore, the polycarbonates are one of the most promising targets from CO_2_, which will contribute to the incorporation of large amount of CO_2_ into chemicals due to the large market size. Polycarbonates have been industrially produced by using phosgene as a carbonyl source, however, phosgene is highly toxic, and the process gives rise to a large amount of salts by neutralization. To overcome these drawbacks, processes using organic carbonates as a carbonyl source have been developed such as condensation of diols and organic carbonates^40^,^41^,^42^ and ring-opening polymerization of cyclic carbonates^43^,^44^,^45^,^46^,^47^ (Fig. 1). However, these processes have similar problems to the phosgene process because the organic carbonate substrates in these processes have been usually synthesized by the reaction of phosgene with the corresponding alcohols or epoxides, and an alternative environmental-benign synthesis process of the organic carbonates have not been established. As for processes using CO_2_ as a carbonyl source, copolymerization of cyclic ethers and CO_2_ have been intensively investigated (Fig. 1) (selected reviews^48^,^49^,^50^,^51^ and selected recent papers^52^,^53^,^54^,^55^,^56^,^57^,^58^,^59^,^60^,^61^,^62^,^63^,^64^). Epoxides and oxetanes have been used as starting materials; however the copolymerization of CO_2_ and cyclic ethers with five-membered or larger ring has not been reported at all because such cyclic ethers are difficult to be prepared due to the low stability. On the other hand, the direct polymerization of diols and CO_2_ by dehydration condensation will enable the synthesis of polycarbonates containing longer alkyl chains (Fig. 1, this work). However, the dehydration condensation of diols and CO_2_ is seriously limited by the reaction equilibrium. For example, it is well-known that the equilibrium yield of propylene carbonate from 1,2-propanediol and CO_2_ on 1,2-propanediol basis has been estimated to be below 2%^65^. Regarding the direct synthesis of polycarbonates from α,ω-diols and CO_2_, to the best of our knowledge, there are no reports on catalytic and non-catalytic synthesis methods, although conversion of CO_2_, diols and dihalides to polycarbonates using K_2_CO_3_ was reported^66^.

Herein, we demonstrate that the combination of CeO_2_ catalyst and 2-cyanopyridine promoter is effective for the direct copolymerization of diols and CO_2_. This is a first report on the catalytic direct synthesis of cooligomers from CO_2_ and diols.

## Results

### Catalyst screening

**Figure 2:**
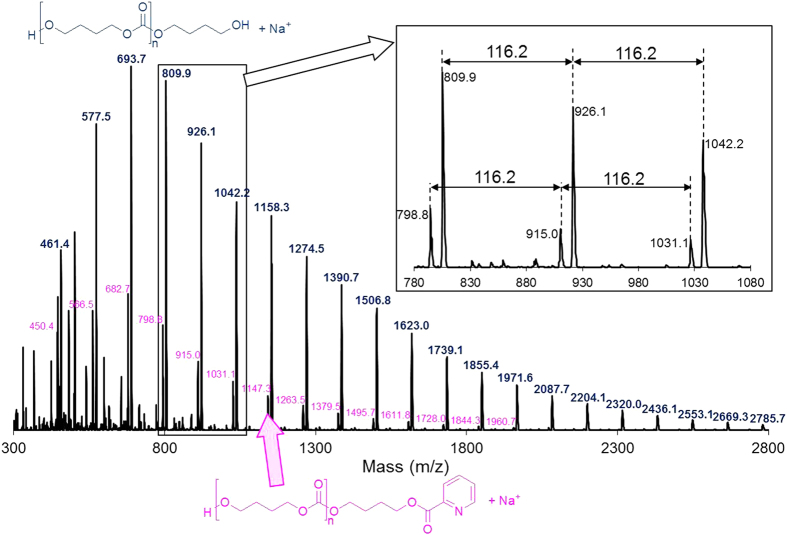
MALDI-TOF mass spectrum of the products from CO_2_ and 1,4-butanediol using CeO_2_ and 2-cyanopyridine.

First, the polymerization from CO_2_ and 1,4-butanediol was investigated using various metal oxides with 2-cyanopyridine (Table 1). 2-Cyanopyridine was selected as a dehydrating agent because 2-cyanopyridine is preferable for the hydration over CeO_2_^33^,^34^,^35^,^67^,^68^. The reaction was carried out with an autoclave reactor containing a metal oxide (0.17 g), 1,4-butanediol (10 mmol), 2-cyanopyridine (100 mmol) and CO_2_ (5.0 MPa) at 403 K. Conversion and selectivity were calculated on the 1,4-butanediol basis. The detailed data for conversion of 2-cyanopyridine are shown in Supplementary Table S1. No oligomer product was obtained without a metal oxide catalyst (Table 1, entry 13). CeO_2_ provided the oligomer in 97% yield (*M*_n_ = 1070, dispersity (*M*_w_/*M*_n_) = 1.33) with slight amount of 4-hydroxybutyl picolinate, which was produced by 2-cyanopyridine and 1,4-butanediol (Table 1, entry 1), and the *M*_n_ of the oligomer corresponds to the oligomers formed from eight CO_2_ and eight 1,4-butanediol. MALDI-TOF mass spectroscopy revealed the formation of the alternating cooligomer from CO_2_ and 1,4-butanediol (Fig. 2), and confirmed no formation of the ether bond. In addition, 2-picolinamide was produced selectively by reaction of 2-cyanopyridine with H_2_O that is produced by the copolymerization from CO_2_ and 1,4-butanediol (Supplementary Table S1). On the other hand, other metal oxides showed lower conversion than CeO_2_ and gave no oligomers (Table 1, entries 3–12). Others would include dimer, trimer or diester produced from 2-cyanopyridine and 1,4-butanediol. Therefore, among the metal oxides tested, CeO_2_ is the only active metal oxide for the reaction by using 2-cyanopyridine as a dehydrating agent. It should be noted that CeO_2_ alone without 2-cyanopyridine provided no oligomer (not shown). Taking this result into consideration, the combination of CeO_2_ and 2-cyanopyridine is essential for the formation of the oligomer of 1,4-butanediol and CO_2_. We first demonstrated direct copolymerization from CO_2_ and 1,4-butanediol using the combination of CeO_2_ catalyst and 2-cyanopyridine promoter. In addition, the reusability of CeO_2_ catalyst was investigated. CeO_2_ was easily retrieved from the reaction mixture by decantation, and the collected catalyst was washed with methanol, followed by calcining at 873 K for 3 h, and then the recovered CeO_2_ was used for the next reaction. CeO_2_ could be reused without remarkable loss of activity and selectivity (Table 1, entry 2), and XRD and BET analyses confirmed that the structure of CeO_2_ was unchanged during the reusability test (Supplementary Fig. S1). In addition, the dissolved amount of Ce species in the filtrate was below the detection level (<0.1 %) of ICP-AES, which indicates that CeO_2_ worked as a truly heterogeneous catalyst in this reaction.

### Performance of combination of CeO_2_ catalyst and 2-cyanopyridine promoter

2-Cyanopyridine reacts with one mole of H_2_O to provide 2-picolinamide, indicating that 10 mmol of 2-cyanopyridine is theoretically necessary to convert all of 1,4-butanediol to the corresponding oligomer when 10 mmol of 1,4-butanediol is used. Effect of 2-cyanopyridine amount was studied using CeO_2_ catalyst (Table 2, the detailed data for conversion of 2-cyanopyridine are shown in Supplementary Table S2). 10 mmol and larger than 10 mmol of 2-cyanopyridine provided almost the same conversion and *M*_n_ (Table 2, entries 2–6), although 5 mmol of 2-cyanopyridine is not effective due to the smaller amount of 2-cyanopyridine than the theoretical amount (Table 2, entry 1). Therefore, the equivalent amount of 2-cyanopyridine is enough for the formation of the oligomer from 1,4-butanediol and CO_2_.

Low CO_2_ pressure is preferable from the environmental and economic viewpoints. The effect of CO_2_ pressure was investigated using CeO_2_ catalyst and 2-cyanopyridine promoter (Table 3, the detailed data for conversion of 2-cyanopyridine are shown in Supplementary Table S3). The reaction proceeds even at low CO_2_ pressure of 0.5 MPa to give the oligomer in good yield (Table 3, entry 1), although the conversion and *M*_n_ gradually decreased with decreasing CO_2_ pressure. This result provides the possibility to perform the reaction at low CO_2_ pressure.

**Figure 3:**
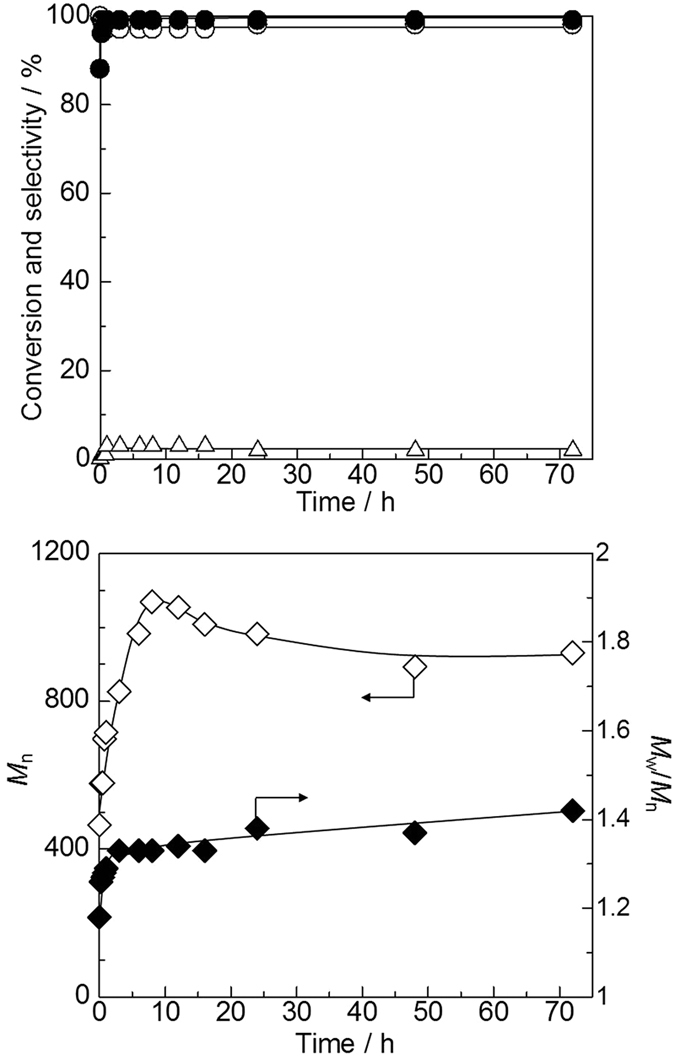
Time-course of direct polymerization of 1,4-butanediol and CO_2_ using CeO_2_ catalyst and 2-cyanopyridine. (**a**) Conversion and selectivity (•: conversion, ○: selectivity to oligomer, ▵: selectivity to 4-hydroxybutyl picolinate. (**b**) *M*_n_ and *M*_w_/*M*_n_ (⋄: *M*_n_, ♦: *M*_w_/*M*_n_). Reaction Conditions: CeO_2_ 0.17 g, 1,4-butanediol 10 mmol, 2-cyanopyridine 100 mmol, CO_2_ 5 MPa (at r.t.), 403 K.

The time-course of the copolymerization of CO_2_ and 1,4-butanediol was investigated using CeO_2_ catalyst and 2-cyanopyridine promoter (Fig. 3, the detailed data are shown in Supplementary Table S4 and Supplementary Figs S2 and S3). The reaction rapidly proceeded to reach 99% conversion in one hour, and the high selectivity to the oligomer (≥97%) was maintained from the short reaction time, which strongly indicates that the oligomers were not produced via formation of tetramethylene carbonate, the corresponding cyclic carbonate. On the other hand, the *M*_n_ increased with the reaction time up to 8 h, but decreased gradually at more than 8 h. Dispersity also increased to about 1.3 in one hour and gradually increased at more than 1 h. The decrease of *M*_n_ and increase of dispersity are attributed to degradation of the oligomers and/or intramolecular termination by the nucleophilic attack of the OH group at the end of polymer, which is known as back-biting^69^,^70^.

Finally, the scope of diols was investigated in the copolymerization of CO_2_ and diols using CeO_2_ catalyst and 2-cyanopyridine promoter (Table 4, the detailed data for conversion of 2-cyanopyridine are shown in Supplementary Table S5). Linear C4–C10 α,ω-diols were converted to the corresponding cooligomers in good yields. The corresponding cyclic carbonates were not also observed in the case of C5–C10 diols, which supports that the direct dehydration condensation of diols and CO_2_ takes place in this reaction system. The average number of the repeating unit of these copolymers was 7 ~ 8 even with any diols. 1,4-Cyclohexanedimethanol and 1,4-benzenedimethanol, which are diols with rigid structure, were converted to the corresponding copolymers, however, the reactivity and *M*_n_ were lower than those of linear alkyl diols. To examine the effect of the position of the OH group, the combination of CeO_2_ catalyst and 2-cyanopyridine promoter was applied to 1,5-hexanediol having one primary and one secondary OH groups, 2,5-hexanediol having two secondary OH groups, and 2,5-dimethyl-2,5-hexanediol having two tertiary OH groups. 1,5-Hexanediol showed lower conversion, selectivity and *M*_n_ than 1,6-hexanediol having two primary OH groups. 2,5-Hexanediol showed further lower conversion, selectivity and *M*_n_ than 1,5-hexanediol. In the case of 2,5-dimethyl-2,5-hexanediol, the corresponding oligomer was not obtained. Therefore, steric hindrance around the OH group drastically decreases the substrate reactivity.

### Proposed reaction mechanism

**Figure 4:**
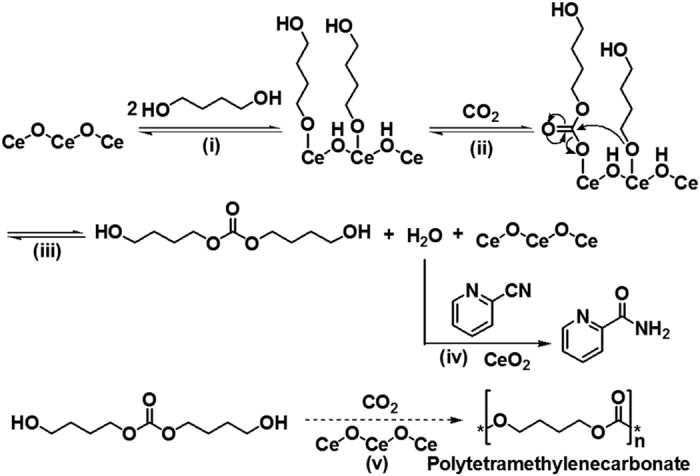
Proposed reaction mechanism of the formation of cooligomers from 1,4-butanediol and CO_2_ using CeO_2_ catalyst and 2-cyanopyridine.

The proposed reaction mechanism is shown in Fig. 4. Based on the previous reports on carbonate synthesis from alcohol and CO_2_ over CeO_2_ catalyst^35^, the reaction starts with (i) adsorption of diol on CeO_2_ surface to form alkoxide adspecies. (ii) CO_2_ insertion to the some alkoxide adspecies, providing some carbonate adspecies. (iii) Nucleophilic attack of the oxygen anion in the alkoxide adspecies to the carbonate adspecies to afford the corresponding carbonate from 1,4-butanediol and H_2_O. (iv) Removal of the produced H_2_O by hydration of 2-cyanopyridine to 2-picolinamide over CeO_2_^33^,^34^,^35^,^67^. (v) Finally, further reaction of the produced carbonate with CO_2_ 1,4-butanediol or produced cooligomer, giving polytetramethylenecarbonate. Among these reaction steps, the step (iv) is very important in the polycarbonate synthesis, which will drastically shift the reaction to the product side by removal of H_2_O from the reaction media.

## Discussion

In summary, we first demonstrated the direct copolymerization from CO_2_ and diols using the combination of CeO_2_ catalyst and 2-cyanopyridine promoter. Various diols including α,ω-diols with long alkyl chain can be transformed to the corresponding cooligomers, which cannot be obtained by the conventional methods with cyclic carbonate, epoxides or oxetanes. This catalyst system will not only open up a new epoch for polymer chemistry, particularly polycarbonate synthesis, but also make a large impact on transformation of CO_2_.

## Methods

### Materials

Preparation of CeO_2_ catalyst was carried out by calcining CeO_2_-HS (Daiichi Kigenso, Japan. The purity of CeO_2_: 99.97%) for 3 h in air at 873 K. The specific surface area (BET method) of the CeO_2_ was 84 m^2^/g. All the chemicals for organic reactions were commercially available and were used without further purification. Other metal oxides were commercially available or synthesized by the precipitation method: ZrO_2_ (Daiichi Kigenso Kogyo Co. Ltd., Zr(OH)_4_ was calcined under air at 873 K for 3 h.), MgO (Ube Industries, Ltd., MgO 500A, 873 K, 3 h), TiO_2_ (Nippon Aerosil Co. Ltd., P-25), γ-Al_2_O_3_ (Nippon Aerosil), ZnO (FINEX-50, Sakai Chemical Industry Co.,Ltd), SiO_2_ (Fuji Silysia Chemical Ltd., 773 K, 1 h), Nb_2_O_5_ (Companhia Brasileira de Metalurgia e Mineracao (CBMM), Nb_2_O_5_∙nH_2_O was calcined at 773 K for 3 h). Y_2_O_3_, La_2_O_3_ and Pr_6_O_11_ were prepared by the precipitation method. Y(NO_3_)_3_∙nH_2_O (Wako Pure Chemical Industries Ltd., >99.9%), La(NO_3_)_3_∙6H_2_O (Wako Pure Chemical Industries Ltd., >99.9%) and Pr(NO_3_)_3_∙nH_2_O (Wako Pure Chemical Industries Ltd., >99.5%) were used as precursors. A precursor (25 g) was dissolved in water (100 ml) and NH_3_aq (1 M) was dropped with stirring until the pH of the solution became 10, resulting in a precipitate. The precipitate was filtered and washed by water, followed by drying at 383 K overnight (12 h) and calcining under air at 873 K (673 K for La_2_O_3_) for 3 h.

### Typical procedure for direct polymerization of CO_2_ and 1,4-butanediol

All the reactions were carried out in an autoclave reactor with an inner volume of 190 mL. The standard procedure of direct polymerization of CO_2_ and 1,4-butanediol using the combination catalyst of CeO_2_ and 2-cyanopyridine was as follows: CeO_2_ (0.17 g, 1 mmol), 1,4-butanediol 0.90 g (10 mmol) and 2-cyanopyridine 10.4 g (100 mmol) were put into the autoclave together with a spinner, and then the reactor was purged with 1 MPa CO_2_ (Shimakyu Co. Ltd., >99.5%) three times. The autoclave was pressurized with CO_2_ to the desired pressure (typically 5.0 MPa) at room temperature, and then the autoclave was heated to 433 K, where the CO_2_ pressure was about 12 MPa. The mixture was constantly stirred during the reaction. After the reaction time, the reactor was cooled in water bath to room temperature. THF (20 ml) was added to the liquid phase as a solvent, and 1-hexanol (~0.2 ml) was also added as an internal standard substance for a quantitative analysis. The reactor was washed with THF, and the liquids used in washing were added to the reaction mixture. Amount of 2-cyanopyridine and products from 2-cyanopyridine such as 2-picolinamide and 4-hydroxybutyl picolinate were analyzed by gas chromatography (Shimadzu GC-2014) equipped with an FID using a CP/Sil 5 CB. Since produced cooligomers is decomposed by heating, the amount of 1,4-butanediol was analyzed by HPLC (Shimazu Prominence) equipped with an RI detector (RID-10A) using a Pheny-Hexyl Luna column (Phenomenex, particle size 5 μm, 250 mm × 4.6 mm, conditions: developing solvent, H_2_O/CH_3_OH = 70/30, 0.5 ml/min, 313 K). Since produced cooligomers were precipitated by addition of the developing solvent (about 20-fold dilution), the precipitated cooligomers were removed by filtration before analyzing by HPLC. This filtration operation was conducted at least two times until the precipitation was not observed. The qualitative analysis of the products were conducted by a gas chromatograph equipped with a quadrupole mass spectrometer (GC-MS, Shimazu QP5050) using the same capillary columns and NMR (Bruker, AV400). The oligomerized products were analyzed by MALDI-TOF mass (Shimazu AXIMA-CFR Plus) using dithranol and NaBr as a matrix and ionization agent, respectively, and size exclusion chromatography (SEC, Shimazu Prominence) with a RI detector (RID-10A) using a Shodex HPLC column KF-805L. The developing solvent is THF (Wako Pure Chemical Industries, >99.5%).

The conversion and selectivity were calculated by the following equations (Eqs 1, 2, 3).


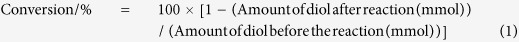



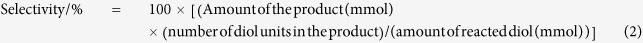



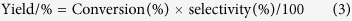


The amount of oligomer was determined by subtraction of the amount of the produced ester from the amount of reacted diol. The products in which the signal was not observed by SEC were assigned to others.

### Catalyst characterization

The surface area of CeO_2_ was measured with BET method (N_2_ adsorption) using Gemini (Micromeritics). X-ray diffraction (XRD) patterns were recorded using MiniFlex 600 with Cu *K*α (40 kV, 15 mA) radiation. The amount of eluted metal into the reaction solution was analyzed by inductively-coupled plasma atomic emission spectrometry (ICP-AES, Thermo Fisher Scientific iCAP 6500).

## Additional Information

**How to cite this article**: Tamura, M. *et al.* Direct Copolymerization of CO_2_ and Diols. *Sci. Rep.* **6**, 24038; doi: 10.1038/srep24038 (2016).

## Supplementary Material

Supplementary Information

## Figures and Tables

**Figure 1:**
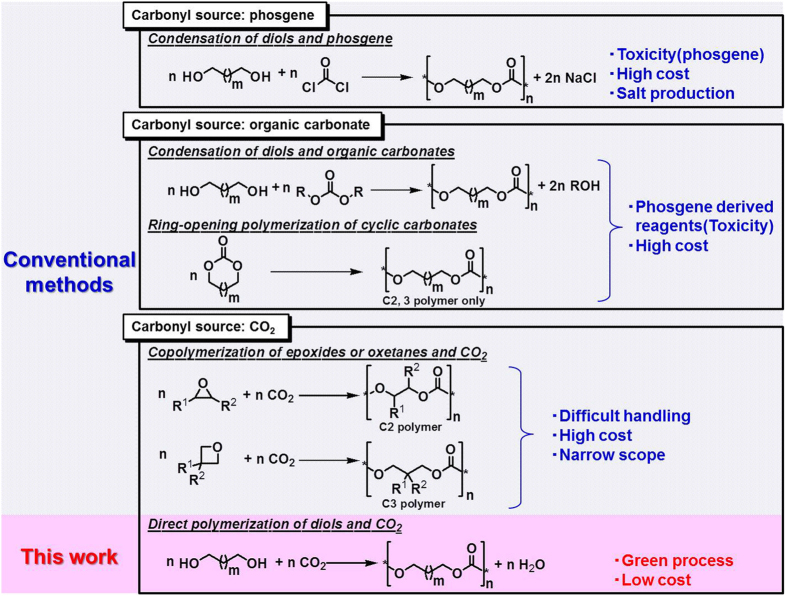
Synthesis methods of polycarbonates.

**Table 1:**
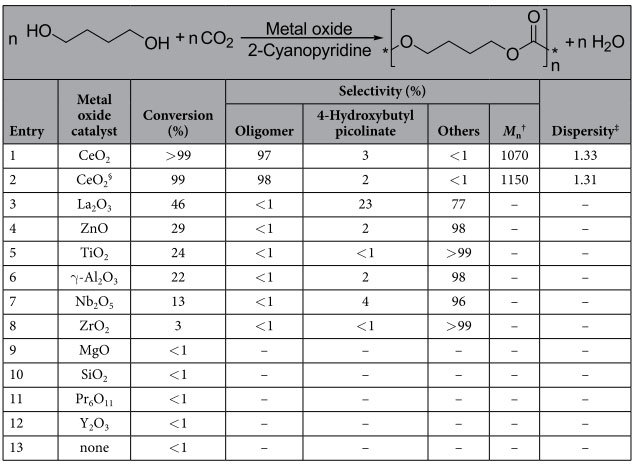
Direct copolymerization of 1,4-butanediol and CO_2_ using a metal oxide catalyst and 2-cyanopyridine^*^.

^*^Reaction conditions: metal oxide 0.17 g, 1,4-butanediol 10 mmol, 2-cyanopyridine 100 mmol, CO_2_ 5 MPa (at r.t.), 403 K, 8 h. ^†^Based on crude sample, polystyrene equivalent molar mass (*M*_n_(SEC)). ^‡^Dispersity is an index (*M*_w_/*M*_n_) determined by SEC with THF as eluent. ^§^Second use.

**Table 2:** Effect of 2-cyanopyridine amount on the polymerization of 1,4-butanediol and CO_2_ using CeO_2_ catalyst*.

Entry	2-Cyanopyridine [mmol]	Conv.[%]	Selectivity (%)			*M*_n_†	Dispersity‡
			Oligomer	4-Hydroxybutyl picolinate	Others		
1	5	68	99	1	<1	510	1.14
2	10	93	98	2	<1	910	1.37
3	20	98	98	2	<1	1110	1.34
4	50	99	98	2	<1	1030	1.32
5	100	99	97	4	<1	1070	1.33
6	200	99	97	3	<1	900	1.33

^*^Conditions: CeO_2_ 0.17 g, 1,4-butanediol 10 mmol, 2-cyanopyridine 5–200 mmol, CO_2_ 5 MPa (at r.t.), 403 K, 8 h.

^†^Based on crude sample, polystyrene equivalent molar mass (*M*_n_(SEC)).

^‡^Dispersity is an index (*M*_w_/*M*_n_) determined by SEC with THF as eluent.

**Table 3:** Effect of CO_2_ pressure on the polymerization of 1,4-butanediol and CO_2_ using CeO_2_ catalyst and 2-cyanopyridine*.

Entry	CO_2_ pressure	Conversion (%)	Selectivity (%)			*M*_n_†	Dispersity‡
			Oligomer	4-Hydroxybutyl picolinate	Others		
1	0.5	88	99	1	<1	630	1.24
2	1	93	99	1	<1	700	1.29
3	2	96	99	1	<1	790	1.31
4	3	98	99	1	<1	900	1.32
5	5	99	97	3	<1	1070	1.33

^*^Reaction conditions: CeO_2_ 0.17 g, 1,4-butanediol 10 mmol, 2-cyanopyridine 100 mmol, CO_2_ 0.5–5 MPa (at r.t.), 403 K, 8 h.

^†^Based on crude sample, polystyrene equivalent molar mass (*M*_n_(SEC)).

^‡^Dispersity = polydispersity index (*M*_w_/*M*_n_) determined by SEC with THF as eluent.

**Table 4:** Scope of diols in the direct polymerization of diol and CO_2_ using CeO_2_ catalyst and 2-cyanopyridine*.

Diol	Conversion (%)	Selectivity (%)			*M*_n_‡	Dispersity§
		Oligomer	Ester†	Others		
1,4-Butanediol	>99	97	3	<1	980	1.38
1,5-Pentanediol	99	99	1	<1	930	1.34
1,6-Hexanediol	99	99	1	<1	1080	1.31
1,8-Octanediol	99	99	1	<1	1200	1.33
1,10-Decanediol	94	97	3	<1	1650	1.26
1,4-Cyclohexanedimethanol	41	98	2	<1	510	1.04
1,4-Benzenedimethanol	56	82	11	7	590	1.10
1,5-Hexanediol	93	99	1	<1	530	1.10
2,5-Hexanediol	6	60	2	38	450	1.01
2,5-Dimethyl-2,5-hexanediol	<1	–	–	–	–	–

^*^Conditions: CeO_2_ 0.17 g, diol 10 mmol, 2-cyanopyridine 100 mmol, CO_2_ 5 MPa (at r.t.), 403 K, 24 h.

^†^Ester is formed from 2-cyanopyridine and diol.

^‡^Based on crude sample, polystyrene equivalent molar mass (*M*_n_(SEC)).

^§^Dispersity is an index (*M*_w_/*M*_n_) determined by SEC with THF as eluent.

## References

[b1] Aresta M. Carbon dioxide as chemical feedstock, WILEY-VCH 2010.

[b2] Aresta M. & Dibenedetto A. Utilisation of CO_2_ as a chemical feedstock: opportunities and challenges. Dalton Trans. 28, 2975–2992 (2007). 17622414 10.1039/b700658f

[b3] Dibenedetto A. Angelini A. & Stufano P. Use of carbon dioxide as feedstock for chemicals and fuels: homogeneous and heterogeneous catalysis. J. Chem. Technol. Biotechnol. 89, 334–353 (2014).

[b4] Sakakura T., Choi J.-C. & Yasuda H. Transformation of carbon dioxide. Chem. Rev. 107, 2365–2387 (2007). 17564481 10.1021/cr068357u

[b5] Gomes C. D. N. *et al.* A diagonal approach to chemical recycling of carbon dioxide: organocatalytic transformation for the reductive functionalization of CO_2_. Angew. Chem. Int. Ed. 51, 187–190 (2012). 10.1002/anie.201105516 21960366

[b6] Coates G. W. & Moore D. R. Discrete Metal‐Based Catalysts for the Copolymerization of CO_2_ and Epoxides: Discovery, Reactivity, Optimization, and Mechanism. Angew. Chem. Int. Ed. 43, 6618–6639 (2004). 10.1002/anie.200460442 15558659

[b7] Cokoja M., Bruckmeier C., Rieger B., Herrmann W. A. & Kühn F. E. Transformation of Carbon Dioxide with Homogeneous Transition‐Metal Catalysts: A Molecular Solution to a Global Challenge? Angew. Chem. Int. Ed. 50, 8510–8537 (2011). 10.1002/anie.201102010 21887758

[b8] Shi J. *et al.* Enzymatic conversion of carbon dioxide. Chem. Soc. Rev. 44, 5981–6000 (2015). 26055659 10.1039/c5cs00182j

[b9] Tripp B. C., Smith K. & Ferry J. G. Carbonic Anhydrase: New Insights for an Ancient Enzyme. J. Bio. Chem. 276, 48615–48618 (2001). 11696553 10.1074/jbc.R100045200

[b10] Merz K. M. Jr. Carbon dioxide binding to human carbonic anhydrase II. J. Am. Chem. Soc. 113, 406–411 (1991).

[b11] Zheng Y. J. & Merz K. M. Jr. Mechanism of the human carbonic anhydrase II-catalyzed hydration of carbon dioxide. J. Am. Chem. Soc. 114, 10498–10507 (1992).

[b12] Kiefer L. L., Paterno S. A. & Fierke C. A. Hydrogen bond network in the metal binding site of carbonic anhydrase enhances zinc affinity and catalytic efficiency. J. Am. Chem. Soc. 117, 6831–6837 (1995).

[b13] Vivier L. & Duprez D. Ceria-Based Solid Catalysts for Organic Chemistry. ChemSusChem 3, 654–678 (2010). 20486156 10.1002/cssc.201000054

[b14] Sahu T., Bisht S. S., Das K. R. & Kerkar S. Nanoceria: Synthesis and biomedical applications. Current Nanoscience 9, 588–593 (2013).

[b15] Wang Y. *et al.* Heterogeneous Ceria Catalyst with Water-Tolerant Lewis Acidic Sites for One-Pot Synthesis of 1,3-Diols via Prins Condensation and Hydrolysis Reactions J. Am. Chem. Soc. 135, 1506–1515 (2013). 23228093 10.1021/ja310498c

[b16] Primo A., Aguado E. & García H. CO_2_-Fixation on Aliphatic α,ω-Diamines to Form Cyclic Ureas, Catalyzed by Ceria Nanoparticles that were Obtained by Templating with Alginate. ChemCatChem 5, 1020–1023 (2013).

[b17] Juárez R., Concepción P., Corma A., Fornes V. & García H. Gold-Catalyzed Phosgene-Free Synthesis of Polyurethane Precursors. Angew. Chem. Int. Ed. 49, 1286–1290 (2010). 10.1002/anie.200905160 20084647

[b18] Juárez R., Concepción P., Corma A. & García H. Ceria nanoparticles as heterogeneous catalyst for CO_2_ fixation by ω-aminoalcohols. Chem. Commun. 46, 4181–4183 (2010). 10.1039/c001955k 20411187

[b19] Tamura M. & Tomishige K. Redox Property at Low Temperature of CeO_2_ for Direct Synthesis of Imine from Alcohol and Amine. Angew. Chem. Int. Ed. 54, 864–867 (2015). 10.1002/anie.201409601 25414058

[b20] Tamura M., Tonomura T., Shimizu K.-i. & Satsuma A. Transamidation of amides with amines under solvent-free conditions using a CeO_2_ catalyst. Green Chem. 14, 717–724 (2012).

[b21] Vernekar A. A., Das T. & Mugesh G. Vacancy-Engineered Nanoceria: Enzyme Mimetic Hotspots for the Degradation of Nerve Agents. Angew. Chem. Int. Ed. 55, 1412–1416 (2016). 10.1002/anie.201510355 26663633

[b22] Tomishige K., Furusawa Y., Ikeda Y., Asadullah M. & Fujimoto K. CeO_2_–ZrO_2_ solid solution catalyst for selective synthesis of dimethyl carbonate from methanol and carbon dioxide. Catal. Lett. 76, 71–74 (2001).

[b23] Tomishige K. & Kunimori K. Catalytic and direct synthesis of dimethyl carbonate starting from carbon dioxide using CeO_2_-ZrO_2_ solid solution heterogeneous catalyst: effect of H_2_O removal from the reaction system. Appl. Catal. A 237, 103–109 (2002).

[b24] Tomishige K. *et al.* Catalytic performance and properties of ceria based catalysts for cyclic carbonate synthesis from glycol and carbon dioxide. Green Chem. 6, 206–214 (2004).

[b25] Yoshida Y., Arai Y., Kado S., Kunimori K. & Tomishige K. Direct synthesis of organic carbonates from the reaction of CO_2_ with methanol and ethanol over CeO_2_ catalysts. Catal. Today, 115, 95–101 (2006).

[b26] Aresta M. *et al.* Influence of Al_2_O_3_ on the performance of CeO_2_ used as catalyst in the direct carboxylation of methanol to dimethylcarbonate and the elucidation of the reaction mechanism. J. Catal. 269, 44–52 (2010).

[b27] Kumar N. *et al.* Synthesis and characterization of solid base mesoporous and microporous catalysts: Influence of the support, structure and type of base metal. Microporous Mesoporous Mater. 152, 71–77 (2012).

[b28] Tamura M., Honda M., Noro K., Nakagawa Y. & Tomishige K. Heterogeneous CeO_2_-catalyzed selective synthesis of cyclic carbamates from CO_2_ and aminoalcohols in acetonitrile solvent J. Catal. 305, 191–203 (2013).

[b29] Tamura M., Noro K., Honda M., Nakagawa Y. & Tomishige K. Highly efficient synthesis of cyclic ureas from CO_2_ and diamines by a pure CeO_2_ catalyst using a 2-propanol solvent. Green. Chem. 15, 1567–1577 (2013).

[b30] Tamura M., Ito K., Nakagawa Y. & Tomishige K. CeO_2_-catalyzed direct synthesis of dialkylureas from CO_2_ and amines. J. Catal. in press. 10.1016/j.jcat.2015.11.015.

[b31] Honda M. *et al.* Tandem Carboxylation‐Hydration Reaction System from Methanol, CO_2_ and Benzonitrile to Dimethyl Carbonate and Benzamide Catalyzed by CeO_2_. ChemCatChem 3, 365–370 (2011).

[b32] Honda M., Sonehara S., Yasuda H., Nakagawa Y. & Tomishige K. Heterogeneous CeO_2_ catalyst for the one-pot synthesis of organic carbamates from amines, CO_2_ and alcohols. Green Chem. 13, 3406–3413 (2011).

[b33] Honda M. *et al.* Ceria-Catalyzed Conversion of Carbon Dioxide into Dimethyl Carbonate with 2-Cyanopyridine ChemSusChem 6, 1341–1344 (2013). 23801598 10.1002/cssc.201300229

[b34] Honda M. *et al.* Direct Cyclic Carbonate Synthesis from CO_2_ and Diol over Carboxylation/Hydration Cascade Catalyst of CeO_2_ with 2-Cyanopyridine. ACS Catal. 4, 1893–1896 (2014).

[b35] Honda M. *et al.* Organic carbonate synthesis from CO_2_ and alcohol over CeO_2_ with 2-cyanopyridine: Scope and mechanistic studies. J. Catal. 318, 95–107 (2014).

[b36] Tamura M., Kishi R., Nakagawa Y. & Tomishige K. Self-assembled hybrid metal oxide base catalysts prepared by simply mixing with organic modifiers. Nat. Commun. 6, 8580 (2015). 26436638 10.1038/ncomms9580 PMC4600743

[b37] Bansode A. & Urakawa A. Continuous DMC Synthesis from CO_2_ and Methanol over a CeO_2_ Catalyst in a Fixed Bed Reactor in the Presence of a Dehydrating Agent. ACS Catal. 4, 3877–3880 (2014).

[b38] Hoepner L. A. *et al.* Urinary concentrations of bisphenol A in an urban minority birth cohort in New York City, prenatal through age 7 years. Environ. Res. 122, 38–44 (2013). 23312110 10.1016/j.envres.2012.12.003 PMC3602210

[b39] Fatch R., Dillert R. & Bahnemann D. Preparation and characterization of transparent hydrophilic photocatalytic TiO_2_/SiO_2_ thin films on polycarbonate. Langmuir, 29, 3730–3739 (2013). 23363048 10.1021/la400191x

[b40] Ono Y. Dimethyl carbonate for environmentally benign reactions. Catal. Today 35, 15–25 (1997).

[b41] Park J. H. *et al.* Preparation of High-Molecular-Weight Aliphatic Polycarbonates by Condensation Polymerization of Diols and Dimethyl Carbonate. Macromolecules 46, 3301–3308 (2013).

[b42] Pokharkar V. & Sivaram S. Poly(alkylene carbonate)s by the carbonate interchange reaction of aliphatic diols with dimethyl carbonate: Synthesis and characterization. Polymer 36, 4851–4854 (1995).

[b43] Carothers W. H., Dorough G. L. & van Natta F. J. Studies of polymerization and ring formation. X. The reversible polymerization of six-membered cyclic esters. J. Am. Chem. Soc. 54, 761–772 (1932).

[b44] Helou M., Miserque O., Brusson J.-M., Carpentier J.-F. & Guillaume S. M. Organocatalysts for the Controlled “Immortal” Ring-Opening Polymerization of Six-Membered-Ring Cyclic Carbonates: A Metal-Free, Green Process. Chem. Eur. J. 16, 13805–13813 (2010). 20945312 10.1002/chem.201001111

[b45] Ling J., Zhu W. & Shen Z. Controlling ring-opening copolymerization of ε-caprolactone with trimethylene carbonate by scandium tris (2, 6-di-tert-butyl-4-methylphenolate). Macromolecules 37, 758–763 (2004).

[b46] Feng J., He F. & Zhuo R. Polymerization of trimethylene carbonate with high molecular weight catalyzed by immobilized lipase on silica microparticles. Macromolecules 35, 7175–7177 (2002).

[b47] Venkataraman S. *et al.* A Simple and Facile Approach to Aliphatic N-Substituted Functional Eight-Membered Cyclic Carbonates and Their Organocatalytic Polymerization. J. Am. Chem. Soc. 137, 13851–13860 (2015). 26456146 10.1021/jacs.5b06355

[b48] Lu X.-B. & Darensbourg D. J. Cobalt catalysts for the coupling of CO_2_ and epoxides to provide polycarbonates and cyclic carbonates. Chem. Soc. Rev. 41, 1462–1484 (2012). 21858339 10.1039/c1cs15142h

[b49] Kemer M. R., Buchard A. & Williams C. K. Catalysts for CO_2_/epoxide copolymerisation Chem. Commun. 47, 141–163 (2011). 10.1039/c0cc02207a 20941400

[b50] Childers M. I., Longo J. M., Van Zee N. J., LaPointe A. M. & Coates G. W. Stereoselective epoxide polymerization and copolymerization. Chem. Rev. 114, 8129–8152 (2014). 25007101 10.1021/cr400725x

[b51] Sugimoto H. & Inoue S. Copolymerization of carbon dioxide and epoxide. J. Polym. Sci. A 42, 5561–5573 (2004).

[b52] Zhang H., Lin X., Chin S. & Grinstaff M. W. Synthesis and Characterization of Poly(glyceric acid carbonate): A Degradable Analogue of Poly(acrylic acid). J. Am. Chem. Soc. 137, 12660–12666 (2015). 26378624 10.1021/jacs.5b07911

[b53] Ohkawara T., Suzuki K., Nakano K., Mori S. & Nozaki K. Facile Estimation of Catalytic Activity and Selectivities in Copolymerization of Propylene Oxide with Carbon Dioxide Mediated by Metal Complexes with Planar Tetradentate Ligand. J. Am. Chem. Soc. 136, 10728–10735 (2014). 25025746 10.1021/ja5046814

[b54] Liu Y., Ren W.-M., He K.-K. & Lu X.-B. Crystalline-gradient polycarbonates prepared from enantioselective terpolymerization of meso-epoxides with CO_2_. Nat. Commun. 5, 5687 (2014). 25477252 10.1038/ncomms6687

[b55] Romain C. & Williams C. K. Chemoselective Polymerization Control: From Mixed‐Monomer Feedstock to Copolymers. Angew. Chem. Int. Ed. 53, 1607–1610 (2014). 10.1002/anie.201309575 PMC4232277 24453135

[b56] Darensbourg D. J. & Wu G.-P. A One‐Pot Synthesis of a Triblock Copolymer from Propylene Oxide/Carbon Dioxide and Lactide: Intermediacy of Polyol Initiators. Angew. Chem. Int. Ed. 52, 10602–10606 (2013). 10.1002/anie.201304778 23946153

[b57] Nakano K., Kobayashi K., Ohkawara T., Imoto H. & Nozaki K. Copolymerization of epoxides with carbon dioxide catalyzed by iron–corrole complexes: synthesis of a crystalline copolymer. J. Am. Chem. Soc. 135, 8456–8459 (2013). 23713519 10.1021/ja4028633

[b58] Liu Y., Ren W.-M., Liu J. & Lu X.-B. Asymmetric Copolymerization of CO_2_ with meso-Epoxides Mediated by Dinuclear Cobalt (III) Complexes: Unprecedented Enantioselectivity and Activity. Angew. Chem. Int. Ed. 52, 11594–11598 (2013). 10.1002/anie.201305154 24019292

[b59] Lehenmeier M. W. *et al.* Flexibly tethered dinuclear zinc complexes: a solution to the entropy problem in CO_2_/epoxide copolymerization catalysis? Angew. Chem. Int. Ed. 52, 9821–9826 (2013). 10.1002/anie.201302157 23873829

[b60] Zhang H. & Grinstaff M. W. Synthesis of atactic and isotactic poly(1,2-glycerol carbonate)s: degradable polymers for biomedical and pharmaceutical applications. J. Am. Chem. Soc. 135, 6806–6809 (2013). 23611027 10.1021/ja402558m

[b61] Wu G.-P., Darensbourg D. J. & Lu X.-B. Tandem Metal-Coordination Copolymerization and Organocatalytic Ring-Opening Polymerization via Water To Synthesize Diblock Copolymers of Styrene Oxide/CO_2_ and Lactide. J. Am. Chem. Soc. 134, 17739–17745 (2012). 23016983 10.1021/ja307976c

[b62] Kember M. R. & Williams C. K. Efficient magnesium catalysts for the copolymerization of epoxides and CO_2_; using water to synthesize polycarbonate polyols. J. Am. Chem. Soc. 134, 15676–15679 (2012). 22971183 10.1021/ja307096m

[b63] Darensbourg D. J. & Wilson S. J. Synthesis of Poly(indene carbonate) from Indene Oxide and Carbon Dioxide- A Polycarbonate with a Rigid Backbone. J. Am. Chem. Soc. 133, 18610–18613 (2011). 22023460 10.1021/ja208711c

[b64] Nakano K., Hashimoto S., Nakamura M., Kameda T. & Nozaki K. Stereocomplex of Poly(propylene carbonate): Synthesis of Stereogradient Poly(propylene carbonate) by Regio- and Enantioselective Copolymerization of Propylene Oxide with Carbon Dioxide. Angew. Chem. Int. Ed. 50, 4868–4871 (2011). 10.1002/anie.201007958 21374773

[b65] Tomishige K. *et al.* Catalytic performance and properties of ceria based catalysts for cyclic carbonate synthesis from glycol and carbon dioxide. Green Chem. 6, 206–214 (2004).

[b66] Oi S., Nemoto K., Matsuno S. & Inoue Y. Direct synthesis of polycarbonates from CO_2_, diols, and dihalides. Macromol. Rapid. Commun. 15, 133–137 (1994).

[b67] Tamura M., Wakasugi H., Shimizu K.-i. & Satsuma A. Efficient and Substrate‐Specific Hydration of Nitriles to Amides in Water by Using a CeO_2_ Catalyst. Chem. Eur. J. 17, 11428–11431 (2011). 21953923 10.1002/chem.201101576

[b68] Tamura M., Sawabe K., Tomishige K., Satsuma A. & Shimizu K.-i. Substrate-Specific Heterogeneous Catalysis of CeO_2_ by Entropic Effects via Multiple Interactions. ACS Catal. 5, 20–26 (2015).

[b69] Luinstra G. A. *et al.* B. On the formation of aliphatic polycarbonates from epoxides with chromium (III) and aluminum (III) metal–salen complexes. Chem, Eur. J. 11, 6298–6314 (2005). 16106457 10.1002/chem.200500356

[b70] Nakano K., Kamada T. & Nozaki K. Selective Formation of Polycarbonate over Cyclic Carbonate: Copolymerization of Epoxides with Carbon Dioxide Catalyzed by a Cobalt(III) Complex with a Piperidinium End-Capping Arm. Angew. Chem. Int. Ed. 45, 7274–7277 (2006). 10.1002/anie.200603132 17024699

