# Supplementary material for: Direct Copolymerization of CO2 and Diols
Source: Sci Rep. 2016 Apr 14;6:24038. doi: 10.1038/srep24038 (PMC4831014; doi:10.1038/srep24038)
Supplement: Supplementary Information [file srep24038-s1.doc]

**Supporting information**

Direct Copolymerization of CO_2_ and Diols

Masazumi Tamura^1,2^*, Kazuki Ito^1^, Masayoshi Honda^1^, Yoshinao Nakagawa^1^, Hiroshi Sugimoto^3^ and Keiichi Tomishige^1^*

^1^Graduate School of Engineering, Tohoku University, Aoba 6-6-07, Aramaki, Aoba-ku, Sendai, 980-8579, Japan

^2^JST, PRESTO, 4-1-8, Honcho, Kawaguchi, Saitama, 332-0012, Japan.

^3^Department of Industrial Chemistry, Faculty of Engineering, Tokyo University of Science, 12-1 Ichigaya-Funagawara, Shinjuku, Tokyo 162-0826 (Japan)

E-mail: [mtamura@erec.che.tohoku.ac.jp](mailto:mtamura@erec.che.tohoku.ac.jp), [tomi@erec.che.tohoku.ac.jp](mailto:tomi@erec.che.tohoku.ac.jp)

Supplementary Table S1. Details of selectivities on 2-cyanopyridine basis in copolymerization of 1,4-butanediol and CO_2_ using a metal oxide catalyst and 2-cyanopyridine^*^

| Metal oxide  catalyst | Conversion of  2-cyanopyridine [%] | Selectivity on 2-cyanopyridine basis [%]  [%] | | |
| --- | --- | --- | --- | --- |
|  |  | 2-Picolinamide | 4-Hydroxybutyl picolinate | Others |
| CeO_2_ | 9 | 92 | 4 | 4 |
| ZnO | 6 | 8 | 2 | 90 |
| La_2_O_3_ | 4 | 29 | 24 | 47 |
| TiO_2_ | 2 | 82 | <1 | 18 |
| γ-Al_2_O_3_ | 2 | 87 | 2 | 11 |
| Nb_2_O_5_ | <1 | - | - | - |
| MgO | <1 | - | - | - |
| Y_2_O_3_ | <1 | - | - | - |
| ZrO_2_ | 1 | 60 | <1 | 40 |
| Pr_6_O_11_ | 2 | 24 | <1 | 76 |
| SiO_2_ | <1 | - | - | - |
| none | <1 | - | - | - |

*Reaction Conditions: metal oxide 0.17 g, 1,4-butanediol 10 mmol, 2-cyanopyridine 100 mmol, CO_2_ 5 MPa (at r.t.), 403 K, 8 h.

Supplementary Table S2. Details of selectivities on 2-cyanopyridine basis in copolymerization of 1,4-butanediol and CO_2_ using CeO_2_ catalyst and various amount of 2-cyanopyridine^*^

| 2-Cyanopyridine  [mmol] | Conversion of  2-cyanopyridine [%] | Selectivity on 2-cyanopyridine basis [%]  [%] | | |
| --- | --- | --- | --- | --- |
|  |  | 2-Picolinamide | 4-Hydroxybutyl picolinate | Others |
| 5 | 99 | 87 | <1 | 13 |
| 10 | 87 | 89 | 2 | 10 |
| 20 | 46 | 94 | 2 | 4 |
| 50 | 18 | 98 | 2 | <1 |
| 100 | 9 | 92 | 4 | 4 |
| 200 | 6 | 69 | 2 | 29 |

*Reaction Conditions: CeO_2_ 0.17 g, 1,4-butanediol 10 mmol, 2-cyanopyridine 5-200 mmol, CO_2_ 5 MPa (at r.t.), 403 K, 8 h.

Supplementary Table S3 Details of selectivities on 2-cyanopyridine basis in copolymerization of 1,4-butanediol and CO_2_ at various CO_2_ pressure using CeO_2_ catalyst and 2-cyanopyridine^*^

| *P*_CO2_  [MPa] | Conversion of  2-cyanopyridine [%] | Selectivity on 2-cyanopyridine basis [%]  [%] | | |
| --- | --- | --- | --- | --- |
|  |  | 2-Picolinamide | 4-Hydroxybutyl picolinate | Others |
| 0.5 | 7 | 96 | 1 | 3 |
| 1 | 8 | 95 | 1 | 4 |
| 2 | 8 | 98 | 1 | 1 |
| 3 | 9 | 97 | 2 | 2 |
| 5 | 9 | 92 | 4 | 4 |

*Reaction Conditions: CeO_2_ 0.17 g, 1,4-butanediol 10 mmol, 2-cyanopyridine 100 mmol, CO_2_ 0.5-5 MPa (at r.t.), 403 K, 8 h.

Supplementary Table S4 Details data of time-course in copolymerization of 1,4-butanediol and CO_2_ using CeO_2_ catalyst and 2-cyanopyridine: Selectivities on 2-cyanopyridine basis^*^

| *t*  [h] | Conversion of  2-cyanopyridine [%] | Selectivity on 2-cyanopyridine basis [%]  [%] | | |
| --- | --- | --- | --- | --- |
|  |  | 2-Picolinamide | 4-Hydroxybutyl picolinate | Others |
| 0 | 6 | 92 | 0 | 8 |
| 0.25 | 7 | 96 | 1 | 3 |
| 0.5 | 7 | 97 | 2 | 1 |
| 0.75 | 8 | 94 | 2 | 5 |
| 1 | 9 | 86 | 3 | 11 |
| 3 | 9 | 92 | 3 | 4 |
| 6 | 9 | 95 | 3 | 2 |
| 8 | 9 | 92 | 4 | 4 |
| 12 | 10 | 94 | 3 | 3 |
| 16 | 10 | 91 | 3 | 6 |
| 24 | 10 | 90 | 2 | 8 |
| 48 | 10 | 80 | 2 | 18 |
| 72 | 9 | 94 | 2 | 4 |

*Reaction conditions: CeO_2_ 0.17 g, 1,4-butanediol 10 mmol, 2-cyanopyridine 100 mmol, CO_2_ 5 MPa (at r.t.), 403 K.

Supplementary Table S5 Details of selectivities on 2-cyanopyridine basis in copolymerization of various diols and CO_2_ using CeO_2_ catalyst and 2-cyanopyridine^*^

| Diol | Conversion of  2-cyanopyridine [%] | Selectivity on 2-cyanopyridine basis [%]  [%] | | |
| --- | --- | --- | --- | --- |
|  |  | 2-Picolinamide | Monoester from  diol and 2-cyanoppyridine | Others |
| 1,4-Butanediol | 10 | 90 | 2 | 8 |
| 1,5-Pentanediol | 9 | 90 | 1 | 9 |
| 1,6-Hexanediol | 9 | 89 | 1 | 9 |
| 1,8-Octanediol | 9 | 91 | 1 | 8 |
| 1,10-Decanediol | 9 | 88 | 3 | 9 |
| 1,4-Cyclohexanedimethanol | 4 | 80 | 3 | 18 |
| 1,4-Benzenedimethanol | 8 | 67 | 10 | 24 |
| 1,5-Hexanediol | 6 | 93 | 2 | 5 |
| 2,5-Hexanediol | 4 | 54 | 1 | 45 |
| 2,5-Dimethyl-2,5-hexanediol | 2 | 99 | 1 | 0 |

*Reaction conditions: CeO_2_ 0.17 g, diol 10 mmol, 2-cyanopyridine 100 mmol, CO_2_ 5 MPa (at r.t.), 403 K, 8 h.

Supplementary Figure S1. XRD patterns of (a) fresh CeO_2_ and (b) CeO_2_ after the reaction.

Supplementary Figure S2. HPLC charts of the products at various reaction times in Figure 3.

Supplementary Figure S3. SEC charts of the products at various reaction times in Figure 3. The number in the parenthesis is the yield of cooligomers.
